# The role of mesh technology with tumor prosthesis reconstruction to reconstruct the extensor mechanism of knee joint after resection of proximal tibial tumors

**DOI:** 10.1186/s13018-019-1105-1

**Published:** 2019-02-26

**Authors:** Bin Liu, Jia Chang Tan, Hui Lin Wang, Zhenjie Wu, Zhen Chao Yuan, Chang Yuan Wei

**Affiliations:** 1 Department of Bone and Soft Tissue, Affiliated Tumor Hospital of Guangxi Medical University, 71 He Di Road, Nanning, 530021 Guangxi People’s Republic of China; 2 Department of Medical Oncology, Affiliated Tumor Hospital of Guangxi Medical University, 71 He Di Road, Nanning, 530021 Guangxi People’s Republic of China; 3 Department of Breast Tumor Surgery, Affiliated Tumor Hospital of Guangxi Medical University, 71 He Di Road, Nanning, Guangxi 530021 People’s Republic of China

**Keywords:** Proximal tibial tumors, Extensor mechanism, Patellar tendon, Synthetic mesh

## Abstract

**Purpose:**

The aim of this study was to evaluate the role of mesh technique in the reconstruction of the extensor mechanism after resection of proximal tibial tumors.

**Methods:**

We retrospectively analyzed the cases of 14 patients who were diagnosed with proximal tibial tumors at our center and reconstructed with tumor prosthesis, gastrocnemius muscle, and mesh between 2012 and 2017. The treatment strategies for patellar tendon reconstruction primarily involve gastrocnemius reconstruction to cover the tumor prosthesis and mesh reconstruction for the patellar ligament.

**Results:**

Among the 14 patients, the mean was 1.57° (range 0–12°) for active extension versus 105.00° (range 80–120°) for active flexion. The mean for passive extension was 0°. The passive flexion mean was 115.00° (range 90–120°). The extensor lag averaged 1.57° (range 0–12°), and the mean Musculoskeletal Tumor Society score (MSTS) was 23.57 (range 19–27). The average follow-up for all patients was 23.50 months (range 14–37). During the recent follow-up, all patients were able to walk without crutches. Two patients underwent above-the-knee amputation for local recurrence of the tumor, and lung metastasis occurred in three patients after operation. There were no postoperative complications.

**Conclusions:**

Extensor lag was remarkably reduced in the surgery group in comparison to previous study reports. Surgical resection is a simple, reliable, and effective method to remove and control the tumor. Mesh reconstruction of patellar ligament is effective to reconstruct the extensor mechanism of the knee after excision of tumor.

## Introduction

The proximal tibia is the second most common site of primary bone tumors [8]. With the development of surgery and chemotherapy, limb salvage has gradually become the standard treatment for primary malignant bone tumors. Wide or radical surgical margin has been associated with a significant decrease in local tumor recurrence and increased survival rates. Tumor prosthesis reconstruction has recently become an essential approach in the proximal tibia. Major complications after surgery include extensor lag owing to resection of the patellar tendon and infection due to soft tissue defects. The application of gastrocnemius flaps effectively reduces the infection of tumor prostheses. Many reconstruction methods for knee-extension devices in proximal tibial tumor prosthesis replacement have been reported, and there is no accepted gold standard at this time. The goal of this study was to evaluate the function of knee extension after the use of mesh technique at our center.

## Materials and methods

Between 2012 and 2017, patients received tumor prosthesis replacement (LDK or CHUN LI Orthopedics, Beijing, China) after excision of bone and soft tissue tumors. The relevant data from a retrospective study were collected from our medical records information system. Written consent was obtained from the Ethics Committee of the Institute before the initiation of this study. The average patient age was 19.64 years (range 11–54 years), and the study population consisted of eight male and six female patients. The surgical indications for prosthesis replacement were as follows: osteosarcoma (12 patients), MFH of the bone (1 patient), and GCT of the bone (1 patient). Primary bone tumors were also classified according to Enneking stage [9]: IIa (3 patients) and IIb (10 patients). All patients were treated with proximal tibial tumor resection, tumor prosthesis replacement, and tibial extensor reconstruction. According to preoperative or postoperative biopsy pathology, malignant bone tumors were treated with adjuvant chemotherapy. The minimum follow-up time for surviving patients was 1 year. Passive and active range of motion (ROM), extensor lag, Musculoskeletal Tumor Society score (MSTS) score, and complications were recorded for 12 weeks postoperatively.

### Surgical technique

**Fig. 1:**
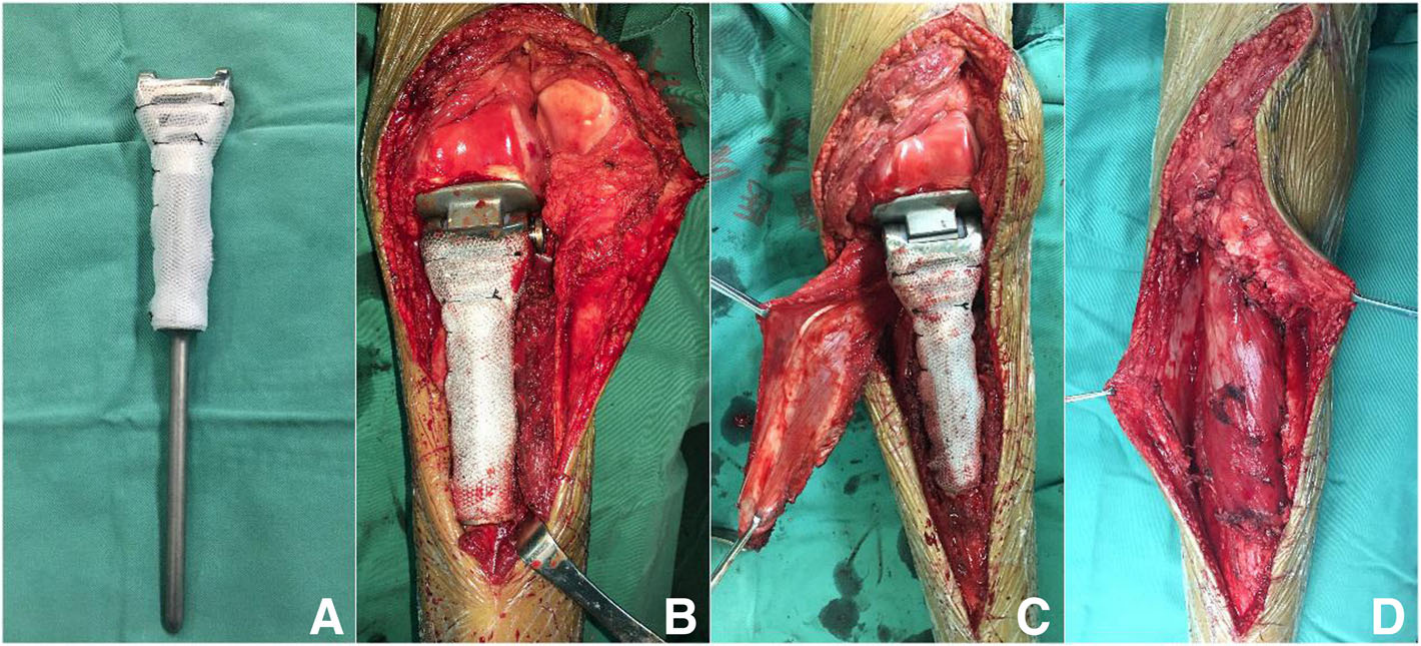
**a** Mesh wrapping the proximal tibia prosthesis. **b** Tumor prosthesis replacement after resection of proximal tibial tumor. **c** Medial head of gastrocnemius muscle covering proximal tibial prosthesis. **d** Reconstruction of extensor mechanism of proximal tibial tumor prosthesis

All operations were performed by experienced surgeons. All patients underwent surgical biopsy before surgery for pathological diagnosis. The operation was performed using medial or lateral incision of the knee joint; the previous biopsy incision was used and extended for implantation of the proximal tibial prosthesis. All patients underwent surgical reconstruction using a tumor prosthesis reconstruction system. Before surgery, the treating surgeon detailed the operative treatment plan, including the treatment of soft tissue and muscle in order to obtain a wide surgical margin under the guidance of MRI. Intra-articular resection and patellar tendon resection were performed in each patient. Proximal tibial osteotomy was performed more than 3 cm from proximal tibial tumors based on preoperative MRI. Reconstructions of the tumor prosthesis, patellar tendon, and gastrocnemius flap were performed in the tibia after osteotomy. Synthetic mesh fixed at the proximal tibia was used to wrap the tumor prosthesis. Reconstruction of the patellar tendon was implemented using synthetic mesh (Bard Crurasoft Patch, 10 cm × 15 cm) to strengthen the fixation at the proximal end of the tibia. Finally, a gastrocnemius flap and synthetic mesh were used to cover the prosthesis. The skin was sutured or a flap transplanted if necessary (Fig. 1).

### Postoperative management

The knee joint of the affected limb was fixed with an extension brace for 6 weeks after operation. The drain was removed 48 to 72 h postoperatively. All patients received continuous passive motion (CPM) at 1 week after operation. Passive ROM started after 2 weeks of treatment. Postoperative adjuvant chemotherapy and weight bearing began 3 weeks after surgery if there was no infection. Partial axial weight-bearing exercise and quadricep contraction were carried out after operation for 6 weeks with an orthopedic brace. All patients underwent radiographic examination follow-ups every 3 months after surgery for 3 years.

## Results

Knee extensor mechanism in all knees was reconstructed using a synthetic mesh to strengthen them after tumor resection. Postoperative knee function was evaluated using active and passive extension and flexion. The mean degree of active extension was 1.57 (range 0–12°), and the mean degree of active flexion was 105.00 (range 80–120°). The mean range of motion for the knee joint was 0° for passive extension, 115.00° (range 90–120°) for passive flexion, 1.57 (range 0°–12°) for extensor lag, and 23.57 (range 19–27) points for MSTS score at 3 months postoperatively. After a median follow-up of 23.50 (range 14–37) months, two patients developed local recurrence and required amputation, and three patients developed lung metastases. No relevant surgery-related complications were noted (Table 1).

**Table 1:** Patient basic data and related clinical data

Case	Sex	Age	Stage	Diagnosis	A-extension	A-flexion	P-extension	P-flexion	Extensor lag	MSTS-score
1	M	14	IIB	Osteosarcoma	0	80	0	90	0	19
2	F	10	IIB	Osteosarcoma	12	120	0	120	12	20
3	M	28	IIB	Osteosarcoma	0	110	0	110	0	21
4	F	15	IIB	Osteosarcoma	0	110	0	110	0	23
5	M	17	IIB	Osteosarcoma	0	90	0	110	0	25
6	F	14	IIB	Osteosarcoma	0	100	0	120	0	25
7	M	16	IIA	Osteosarcoma	0	100	0	110	0	24
8	F	14	IIB	Osteosarcoma	0	90	0	110	0	22
9	M	12	IIB	Osteosarcoma	0	110	0	120	0	22
10	M	16	IIA	Osteosarcoma	0	100	0	110	0	26
11	F	18	IIB	Osteosarcoma	0	110	0	120	0	27
12	M	54	IIA	MFH of bone	10	120	0	120	10	24
13	F	11	IIB	Osteosarcoma	0	110	0	120	0	25
14	M	36	–	GCT of bone	0	120	0	120	0	27

*M* male, *F* female, *MFH* malignant fibrous histiocytoma, *GCT* giant cell tumor, *A* active, *P* passive, *MSTS* Musculoskeletal Tumor Society score

## Discussion

In the past few decades, with the advancement of surgical techniques, tumor prosthesis replacement has been increasingly accepted by orthopedic oncology surgeons [19], and limb salvage therapy has gradually become a mainstream approach. However, postoperative complications—such as aseptic loosening of the prosthesis, prosthesis rupture, infection, and dislocation—are reportedly higher after tumor prosthesis replacement [7, 18]. The most urgent problem after resection of a proximal tibial tumor is the reconstruction of the extensor mechanism and the risk of postoperative infection. The transfer of a gastrocnemius muscle flap reduces the risk of postoperative infection and facilitates effective reconstruction of the extensor mechanism (Pedicled Rotational Medial and Lateral Gastrocnemius Flaps: Surgical Technique). However, the application of the above technology is not perfect, and the main concern is the challenge of reconstructing extensor lag. The prevention of the above symptoms has also become a major concern for surgeons. The inadequacy of postoperative extensor function is the driving force for the surgeon to improve his or her surgical technique. This study discusses the role of synthetic mesh with megaprosthesis in the reconstruction of proximal tibial bone tumors.

Tumor prosthesis replacement is a good choice when the tumor is adjacent to the surface of the knee joint. In addition to replacing the affected bones, the proximal tibial megaprosthesis is critical in reducing the risk of postoperative failure and dysfunction by reconstructing the surrounding ligament and soft tissue [3]. Prosthesis reconstruction failure has been divided into five categories by Henderson. Soft tissue failure is defined as soft tissue attachment defects requiring reoperation. These fixation failures are mainly due to the fixation failure of the tendon around the articular ligament, resulting in instability of the prosthesis or nonunion of the ligament and prosthesis.

The ideal method for reconstruction of the extensor mechanism is still under debate. Repair methods for the extensor mechanism include direct repair, allogeneic tissue repair, and synthetic ligament repair [5, 16]. Direct repair in clinical applications is widely used to manage this challenging clinical problem. However, direct repair of the patellar tendon should be avoided due to the high risk of postoperative complications [4]. Reconstruction of the patellar ligament often involves shortening the extensor mechanism, which is the main cause of the short contraction of scar tissue after operation; therefore, tension-free repair is necessary. Allogeneic tissue reconstruction is another option, which can restore the knee joint’s active extension and reduce the complications caused by autologous transplantation. The results showed that the two methods were similar in effect. Extensor mechanism reconstruction methods using synthetic grafts include Leeds-Keio ligament [10], Trevira tube [12], LARS [6], and mesh [2, 15]. Use of the mesh technique reduced the failure rate of peripheral soft tissue reconstruction. The traditional reconstruction method is to fix the residual patellar ligament to the tumor prosthesis, which helps preserve knee function but limits knee flexion. In order to overcome the negative effects on flexion, we developed a mesh to reconstruct the knee extension mechanism. In this study, the extensor lag (14.28%) was significantly lower than that reported in previous studies. Bickels et al. reported that full extension to an extension lag of 20° was achieved in 44 patients (78%) [1]. If the patellar ligament is too long, there will be extensor delay, and if it is too short, the flexion of the knee will be limited. Surgical techniques and procedures are simple because no special surgical equipment is required. Like normal collagen, synthetic mesh can serve as a framework for the growth of host tissues and contribute to the formation of ligaments [14]. This study showed that with the use of mesh, tensile strength was not decreased, and biological activity was good.

The successful reconstruction of the knee extension mechanism depends on whether the residual patellar tendon and the tumor prosthesis can be effectively mobilized. Effective fixation requires a stable and firm contact interface. A single interface (between the patellar tendon and the prosthesis) does not achieve final healing, because of the presence of the metal prosthesis interface. At present, there is no ideal solution for the reconstruction of the knee extension mechanism; however, the double interface reconstruction method may achieve strong fixation between the two interfaces, making it a potentially viable choice for the reconstruction of knee extension mechanism. In double interface reconstruction, both the patellar tendon and the composite patch interface can be effectively fixed, and the composite patch and the tumor prosthesis interface are fixed through scar formation. A study by Ichikawa found that the use of mesh for extensor reconstruction after proximal tibial resection is a simple, reliable, and successful method [15]. This study also confirmed the safety of mesh technology; no serious complications occurred after operation. In addition, the medial gastrocnemius flap is the best choice for reconstruction of the extensor mechanism and adequate covering of the prosthesis, which may decrease the risk of infection [13, 17]. One study showed that infection rates were reduced to 16.7% after routine use of the gastrocnemius flap [11].

## Study limitations

This study has several limitations. Our sample population was small; further studies with larger numbers of patients are needed. The follow-up time was also short after reconstruction of the extensor mechanism using a synthetic mesh in the proximal tibia. In addition, cases in which the patellar tendon had been violated at the proximal end of the tibia were not included.

## Conclusion

Compared with other forms of reconstruction technology, synthetic mesh has many clinical advantages, including lower cost, applicability to a wide range of operations, reduced risk of disease transmission, and reduced tendon stretching. Reconstruction of the extensor mechanism via reattachment of the patellar tendon to the prosthesis, reconstruction of the extensor mechanism using the patellar ligament, reinforcement of the prosthesis with mesh, and covering of the prosthesis with a gastrocnemius flap can decrease the occurrence of extension lag and provide good clinical function after proximal tibia endoprosthetic reconstruction. Therefore, the application of mesh technology is a simple, efficient, and effective reconstruction method, with the potential for extensive clinical validation in the future.

## Abbreviations

CPM: Continuous passive motion
GCT: Giant cell tumor
MFH: Malignant fibrous histiotoma
MRI: Magnetic resonance imaging
MSTS: Musculoskeletal Tumor Society score
ROM: Range of motion
